# Barriers and facilitators experienced by people with neurological disabilities: A South African health systems perspective

**DOI:** 10.4102/jphia.v17i1.1768

**Published:** 2026-07-31

**Authors:** Prisha Alakram-Khelawon, Sonti Pilusa, Natalie Benjamin-Damons

**Affiliations:** 1 Department of Physiotherapy, Faculty of Health Sciences, University of the Witwatersrand, Johannesburg, South Africa

**Keywords:** contextual factors, barriers, facilitators, neurological disorders, people with neurological disabilities, rehabilitation, health system

## Abstract

**Background:**

Globally, there has been an increase in neurological disorders such as stroke and spinal cord impairments, which often result in permanent disability and require long-term care. However, there is limited evidence on factors influencing the continued care journey for people with neurological disabilities.

**Aim:**

This study aimed to explore the barriers and facilitators experienced by people with neurological disabilities throughout their care journey.

**Setting:**

This study was conducted in a specialised rehabilitation hospital.

**Methods:**

An explorative qualitative design was used. Semi-structured interviews were conducted with 26 patients with neurological disabilities and 15 health professionals. MAXQDA (2024) software was utilised for thematic data analysis.

**Results:**

Key environmental barriers influencing the care journey included limited or delayed access to care, inaccessible infrastructure and transport, a lack of health resources, financial and employment challenges, and poor support and attitudes. Personal factors were identified as the impact of limitations or restrictions on physical function, the patient’s own beliefs, and self-motivation.

**Conclusion:**

People with neurological disabilities experience numerous barriers that suggest gaps in the health system. Throughout the care journey, there is a need to improve health system responsiveness, service accessibility, treatment quality, and health system governance.

**Contribution:**

Specialised health services and system improvements are recommended for people with neurological disabilities, particularly in rehabilitation care and community integration.

## Introduction

The burden of disability caused by neurological disorders is increasing, and globally, approximately 3.4 billion people are affected by neurological disorders such as stroke and spinal cord impairments (SCI).^1^ In South Africa, the disability-adjusted life years (DALY) for neurological disorders showed a 31% increase from 1990 to 2015.^2^ The prevalence and burden of stroke and SCI have increased over the past 30 years.^3,4^ A national registry for spinal cord data or stroke is not available in South Africa. However, available data show that the Gauteng province in South Africa has a 38.7% prevalence of spinal cord injury among patients admitted for rehabilitation.^5^ It is also reported that stroke is one of the leading causes of disability in South Africa, and approximately 25 000 people die annually from stroke.^6^ This increase is concerning, given that neurological disorders such as stroke and SCI often result in permanent disability and require long-term care. Evidence on factors influencing the entire care pathway for these neurological-related disabilities is limited and requires region-specific research.

The care pathway for people with neurological disabilities (PWNDs), such as stroke and SCI, interfaces and extends across many stages of care. This can also be referred to as the ‘care journey’, which encompasses patients’ experiences in the health system and the support from relevant people and/or organisations over a continuum of care.^7,8^ For people with stroke and SCI, this care journey starts with preventative care, then progresses to the onset of the neurological condition in acute care, rehabilitation care, including inpatient and outpatient rehabilitation, and community reintegration. Care pathways and patient experiences can be influenced by a myriad of diverse factors. For example, enablers of the stroke care pathway may include personal motivation, family support, environmental support, rehabilitation programmes, and assistive devices.^9^ The literature identifies other key enablers for people with SCI to return to work, such as disability adaptation, access to assistive devices, and a supportive work environment post disability.^10^

Conversely, prior studies identified significant barriers in care pathways, including excessive costs of assistive technology devices, limited awareness of available options, and difficulties meeting the criteria for receiving these devices.^11^ Tallqvist et al.^12^ found that the rehabilitation process was primarily limited by delayed access, a lack of health skills, and a lack of health resources. Mohan et al.^13^ reported barriers to community reintegration, such as inaccessible infrastructure, cultural and psychological barriers, and secondary complications. Therefore, a rigid medical model of care is not adequate for an integrated care pathway for patients with long-term conditions such as SCI and stroke.^14^ A preventive biopsychosocial approach aimed at achieving optimal functional outcomes and integration should extend from acute care to community care.^15,16^ Hence, it is important to explore the barriers and facilitators experienced by PWNDs throughout their care journey, to improve health services and optimise their health outcomes.

In South Africa, there is growing evidence of the care pathway for people requiring rehabilitation in general. Barriers identified in isolated parts of the care pathway include limited accessibility to rehabilitation services, a lack of government and social support, negative attitudes, a lack of information, and limited accessibility to transport and the work environment.^17,18^ However, the evidence is not specific to PWNDs over a continuum of care, despite a global increase in neurological disorders such as stroke and SCI. This gap highlights the need for comprehensive studies examining how to improve health system responsiveness, service accessibility, treatment quality, and governance to support long-term care and inclusion of PWNDs. Thus, this study aimed to explore contextual factors (personal and environmental) experienced by PWNDs throughout the care journey. The South African government is gaining momentum in the implementation of the national health insurance.^19,20^ Therefore, region- and condition-specific evidence is required to elevate health services, improve health outcomes, and implement an integrated care journey for PWND.

## Research methods and design

### Research design

An exploratory qualitative study design was used to describe the barriers and facilitators experienced in the care journey of PWND. In addition, recommendations to strengthen the health services for PWNDs were explored. This study was one part of a larger PhD study by the corresponding author.

### Study setting

People requiring comprehensive rehabilitation care, such as SCI and stroke, are admitted to a specialised public rehabilitation Hospital in Gauteng province, which is a densely populated urban region in South Africa.^21^ Statistics in South Africa^22^ have reported that approximately 84% of the population is accessing public healthcare. The 24-h public rehabilitation hospital serves the entire Gauteng province and receives referrals from all levels of care. Therefore, it provided the most suitable setting. The rehabilitation hospital ward registers indicated that 98% of the patients admitted had disabilities related to neurological disorders.

### Study participants

Purposeful sampling was appropriate to ensure coverage of the continued care journey for PWNDs. Therefore, the patient sample consisted of adult inpatients and outpatients with no time limitations since the onset of their neurological disorders. Stroke and SCI are the most common neurological disorders in the adult population at the rehabilitation hospital. All participants were aged 18 years or older and children were excluded. All applicable patients were invited to participate in the study. Expert opinions were sought from the heads of departments (HODs) among the current health professionals at the rehabilitation hospital, including doctors, nurses, physiotherapists, occupational therapists, speech and language therapists, audiologists, pharmacists, dietitians, social workers, and psychologists. These HODs managed professional health departments or, in the case of the operational nursing managers, outpatient departments or hospital wards.

### Procedure

Initially, all participants were provided with standardised information and consent forms for participation in the study, as well as for permission to be audio recorded. Semi-structured interviews were conducted to obtain a comprehensive understanding of the patients and HODs by using similar interview guides with open-ended questions. These participants were asked to describe barriers and facilitators they experienced. In addition, they were asked to recommend service improvements in the care pathway. Demographic and other socio-economic data were also collected. Patient data were further categorised according to assistive devices, conditions, waiting times, length of stay, and duration of disability.

The purpose of the study was explained to the participants in a language that they could understand, and informed consent was obtained. A research assistant was available to assist with the patients and language translation as required. All interviews were face-to-face and audio recorded. Interviews were conducted in a private hospital room. Two pilot interviews were conducted, and these results were deliberated with the co-author, Sonti Pilusa. A strategy was implemented to proceed with the subsequent interviews without material changes to the interview guide. Interviews were conducted until data saturation was reached in consultation with the study supervisors. A field journal was implemented, and all research processes and schedules were documented to meet the requirements of research audits.

### Data analysis

Qualitative data were analysed using a framework analysis.^23^ Interviews were audio recorded and then transcribed verbatim by an independent service provider. The primary researcher and research assistant verified the transcripts. The MAXQDA 24 (Analytics Pro) software was used to code the transcripts and develop themes. Continuous familiarisation and re-reading of the data, along with draft coding, were performed during and after the interviews. Two researchers analysed three transcripts inductively and explored the possible codes. Thereafter, the results were discussed, and an inductive coding framework was developed and used to code the remaining transcripts. Similar codes were then categorised into broader categories. This was verified by using a secondary coder at each stage. The final code book was shared with the supervisors for verification. There was an interpretation of the coding and triangulation of data prior to the write-up of the findings.

### Theoretical frameworks used in data analysis

**FIGURE 1:**
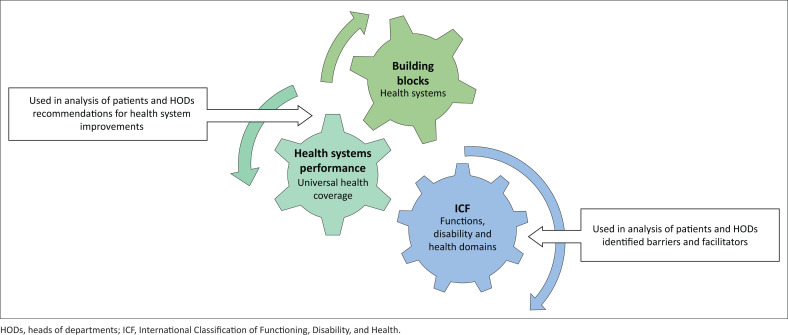
Theoretical frameworks used for data analysis.

Figure 1 refers to the theoretical frameworks used in data analysis as adopted by the World Health Organization (WHO): International Classification of Functioning, Disability, and Health (ICF),^24^ the Building Blocks of the health systems^25^ and the Health System Performance Assessment for universal health coverage.^26^

The emerging categories were classified using the ICF. The ICF conceptualises a biopsychosocial approach to care by optimising health outcomes to improve activities of daily living and maximal participation in life events.^24^ The ICF organises and categorises functioning and disability in key health-related domains of body structure and function, activities, participation, and contextual factors.^27^ The contextual factors comprise of environmental factors (physical, social, and attitudinal environment) as well as personal factors (lifestyle, habits, life events, etc).^28^ The contextual factors identified in this study were further categorised as services, systems and policies, products, technology and man-made environments, support and relationships, attitudes, and other factors.^29^

Participants’ suggestions for health service and systems improvements were categorised and adapted primarily from the Building Blocks of the health systems^25^ and the Health System Performance Assessment for universal health coverage.^26^ While both frameworks describe similar components to categorise and assess the health system, the building blocks provide simple evidence-based components and indicators for health system strengthening. The Health System Performance Assessment for universal health coverage is a modern framework designed to strengthen the health system and advance universal healthcare.

### Methodological rigour

The methodological rigour criteria adhered to the principles of credibility, dependability, confirmability, and transferability.^30,31^ The primary researcher and research assistant were trained and oriented to the study and data-collection materials in advance. An audit trail was maintained, and all the original data are available via the University of the Witwatersrand repository. During data analysis, a code-recode procedure was employed, in which segments of data were coded in separate sessions, and the results were compared. A codebook and field diary were maintained.

The researcher’s position at the hospital is in administrative hospital management. Therefore, rigid reflexivity was practiced, in which the researchers’ personal, professional, and job-related positional views were set aside to prevent bias and maintain neutrality. A research assistant was also allocated to assist with interviews. The patients were not known to the researcher prior to the interview, and the researcher was introduced to them as a physiotherapist. The researcher was known to the HODs; however, they were reassured about the study’s confidentiality and independence. Their participation was voluntary with witnessed informed consent. In addition, participants had the option of being interviewed by a research assistant who was unknown to all patients and HODs.

### Ethical considerations

Ethical clearance to conduct this study was obtained from the University of the Witwatersrand Human Research Ethics Committee (Medical) (No. M220828). The study was registered in the National Health Research Database (GP_202211_037).

## Results

The principal overarching theme that emerged was that various barriers were experienced in the care journey because of inefficiencies in the health system for PWND. Various categories and sub-categories emerged. The results of this study have been outlined in the following subsections:

- Heads of departments and patient demographic information.
- An *overview* of *key* categories, themes, and quot*ations* describing the key barriers and facilitators in the care journey. *An illustrative summary is presented at the end*.
- A summary of suggestions to improve health systems with supporting quotations.

### Demographic information

**FIGURE 2:**
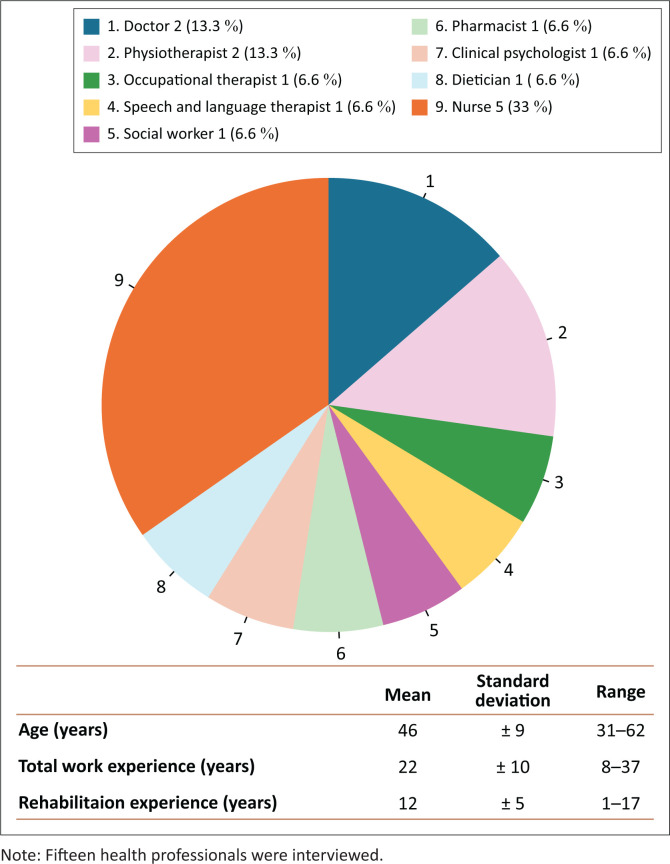
Demographic information of heads of departments.

A total of 41 participants, 26 patients, and 15 HODs were interviewed. Figure 2 outlines the HOD demographics, with an emphasis on years of experience and representation of HODs from all health professions at the hospital. The HODs represented 100% of the departments existing at the rehabilitation hospital under each profession, as well as the Outpatients Department and the operational nursing managers from the various ward categories. The data revealed an in-depth profile of HODs: the mean total years of work experience was 22 years, and the mean years of rehabilitation experience was 12 years.

**FIGURE 3:**
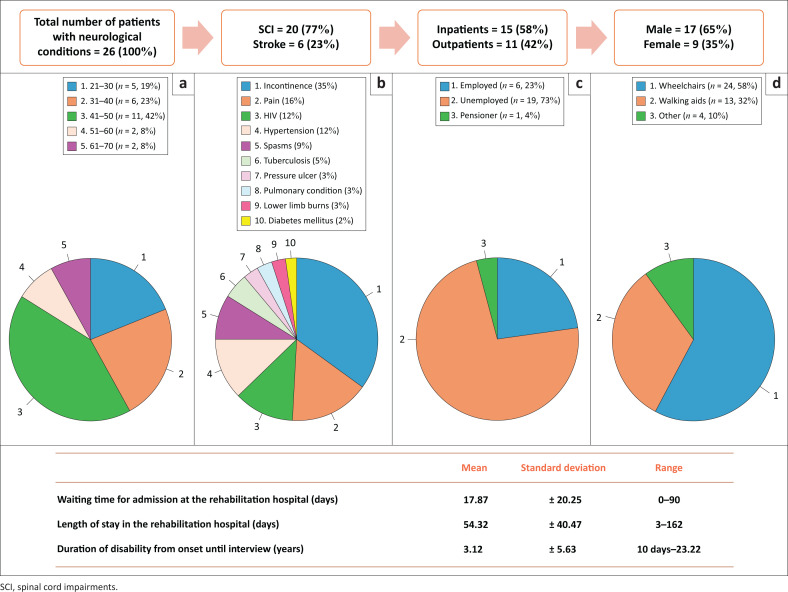
Patients’ demographic information (a) Age (years); (b) Other conditions; (c) Employment status; and, (d) Assistive devices.

A summary of the patients’ demographic information is outlined in Figure 3. There was a lack of access to rehabilitation care, with patient waiting times for admission to rehabilitation recorded at 17.87 days, while the average length of stay in the rehabilitation hospital was 54.32 days. Patients were interviewed between 10 years and 23 years, 22 years post-onset, allowing data to be collected throughout the care journey, from inpatients to outpatients who were integrated into society.

### A Summary of contextual factors experienced in the care pathway

Various barriers and facilitators are outlined in Table 1. These categories and sub-categories of themes identified are based on the findings of the study that were categorised using ICF contextual factors, that is, environmental and personal factors.^24,27,32^

**TABLE 1:** A summary of barriers and facilitators experienced in the care journey.

Contextual factors	Barriers and facilitators
**Enviromental factors** Services, systems and policies	Barriers: Delays and lack of access to care Shortage of health staff A lack of therapeutic services over weekends Poor administration of medication by health staff A lack of patient and caregiver information services Limited community services for PWND Limited financial and employment services and support Facilitators: Positive MDT care received at the rehabilitation hospital Patient and caregiver information services
Products, technology, and the man-made environment	Barriers: Challenges with health resources Challenges with assistive devices A lack of access for PWND in man-made structures A lack of access for PWND in the transport systems
Support and relationships	Barriers: Challenges with homestay A lack of family or caregiver support A lack of community support Facilitators: Peer support Supportive family or caregiver
Attitudes	Barrier: Negative attitude of family, health staff, friends, and community
**Personal factors**	
Physical function	Barrier: Limitations in activity and restrictions in participation because of disability
Beliefs	Barrier: Negative effects of personal beliefs and traditions
Motivation	Barrier: A lack of self-motivation negatively affected patients’ progress in rehabilitation Facilitator: Well-motivated patients positively impacted their progress in rehabilitation care

*Source*: Leonardi M, Lee H, Kostanjsek N, et al. 20 years of ICF – International classification of functioning, disability and health: Uses and applications around the world. Int J Environ Res Public Health. 2022;19(18):11321. https://doi.org/10.3390/ijerph191811321; World Health Organization. ICF checklist, version 2.1a, clinician form [homepage on the Internet]. Geneva; 2003 [cited 2025 Oct 28]. Available from: https://www.who.int/publications/m/item/icf-checklist; Correa CL, Liou TH, Barrios M. Editorial: ICF-based rehabilitation for neurological disease. Front Rehabil Sci. 2022;3:995070. https://doi.org/10.3389/fresc.2022.995070.

PWND, people with neurological disabilities; MDT, multidisciplinary team.

#### Environmental factors: Services, systems and policies

The majority of barriers identified by the participants were categorised as services, systems, and policies.

The participants reported a lack of access to care, as there were delays in acute care and referral to rehabilitation care, resulting in complications and delays in treatment, such as surgical intervention:

‘The hospital told me I have a TB spine, and I had to wait for the operation.’ (P2, SCI, male, 41 years old)

‘… the delay in referral whereby a patient is being kept at a certain hospital … that other hospital will not manage a patient while they are still awaiting the referral [*to rehabilitation*] … already the patient has developed complications …’ (HOD 10, Ola, female, 33 years old)

A shortage of health staff was primarily reported by HODs, which contributed to increased waiting times and shorter treatment times:

‘… we don’t have enough staff from the queue marshals … the doctors and you know do many procedures … but it’s only one person who is doing that so they have to wait and that will be increasing the waiting period also.’ (HOD 3, Ivy, female, 55 years old)

Patients indicated a lack of therapeutic services over the weekend at the rehabilitation hospital:

‘… Weekends there is nothing happening. I think at least they should leave some training facilities just open for us … Just to keep our minds busy … now you just eat and watch TV.’ (P17, Stroke, male, 45 years old)

Participants described poor administration of medication that resulted in taking incorrect medication and there was poor management of the medication inventory:

‘… they did not help me with anything. Instead, I drank the medication, the wrong medication for the long time …’ (P26, SCI, female, 44 years old)

Participants reported challenges in meeting financial obligations. The evidence suggests that social grants were inadequate to cover their medical and living expenses in the care pathway for PWND:

‘… the social worker also helped me by organising things … applying for the social grants … I was also stressed with finances, since I am no longer working, and I will be needing money for many things since I am in a wheelchair …’ (P13, SCI, male, 24 years old)

Various patients face challenges and uncertainty of employment post disability because of limited vocational rehabilitation services, limited accessible transport, and the lack of adaptability in the workplace:

‘… He was operating a carwash … but after his injury, he couldn’t operate it anymore.’ (P4, SCI, male, 26 years old)

‘… to get other jobs we are struggling … you can get a job somewhere … to reach you must use two or three taxis a day … so you just give up.’ (P21, SCI, male, 46 years old)

Although the lack of patient and caregiver information services was reported as a barrier by some patients, HODs also indicated that it can be a facilitator:

‘… the difference between rinsing my bladder … I was just not informed … it made a significant difference … I got a lot less infections … I do not think there is a worked-out programme, a standard presentation … I could have known this three years ago …’(P22, SCI, male, 49 years old)

Heads of departments reported a lack of community services for PWND, especially in primary healthcare:

‘… Our primary healthcare facilities are not equipped to deal with disabilities. They do not have the medication, they do not have … Coloplast … dressings for bed sores … the Primary Health Care facilities are not equipped with consumables, and then with actual experience working with the patient’s condition.’ (HOD 9, Aya, female, 31 years old)

The multidisciplinary team (MDT) care received at the rehabilitation hospital was described by the majority of participants as facilitators:

‘… coming here helped me a lot to be able to be independent … transferring … from wheelchair to the bed and to the bathroom or to the toilet … to take care of myself …’ (P13, SCI, male, 24 years old)

‘… They treat us well … They properly communicate … I feel as if I am at home … the hospital assisted me on walking … I started speaking … my hand it is becoming much better …’ (P1, Stroke, female, 50 years old)

#### Environmental factors: Products, technology, and the man-made environment

Participants, especially the HODs, repeatedly reported a shortage of medical and rehabilitation resources, such as challenges with assistive devices, availability of medication, and a shortage of equipment:

‘… there is a lack of supplies … we do not get the wheelchairs in times or the dressings in times … now the wound that was supposed to heal in a weeks’ time, it takes three months.’ (HOD 4, Ella, female, 62 years old)

‘… the machine which pulls you up … it supports you, like standing … we will have to wait for another … person to finish. Then that is when another person comes in, then I will have to wait for another day to, to be on the machine …’ (P6, SCI, male, 25 years old)

The participants mentioned how inaccessible man-made infrastructure and transport limited access to healthcare, activities, and participation:

‘It changed because now I am using a wheelchair. There are some places that are not accessible for wheelchairs …’ (P21, SCI, male, 46 years old)

‘… First time you move from here to go home, someone picks you up, not public transport, then you are supposed to come here on your own. Then that is when you will see the challenges.’ (P20, SCI, male, 49 years old)

#### Environmental factors: Support and relationships

Many patients verbalised challenges with homestay pre- and post-rehabilitation care:

‘… I have my own caregiver, there are challenges … I have to beg … repeat the same things, so that itself is mostly challenging …’ (P7, SCI, male, 32 years old)

Caregiver and/or family support was reported as a barrier, but if patients had strong family or caregiver support, it was largely a facilitator:

‘… some families would be a barrier because we find that family are not even prepared to take the patient home, so they really feel isolated …’ (HOD 6, Tumi, female, 47 years old)

‘… If you have people who love and support you, you feel like you own the world … But if you do not get support, it is where you have those stress kind of things. Lucky enough I was having a good family.’ (P21, SCI, male, 46 years old)

The patients and HODs described peer support as a facilitator. Peer support was reported as physical or emotional assistance from other patients with similar disabilities:

‘… They put the food there and they go. And you must beg somebody just to feed you … Many times, the patients helped to feed me.’ (P3, SCI, male, 61 years old)

#### Environmental factors: Attitudes

Patients highlighted how the health professional’s negative attitudes affected them in their care pathway:

‘… the Sisters … they don’t have patience like even when she gives you food, … with an attitude … you want to change the TV channel, so we were afraid to ask the nurses …’ (P25, SCI, female, 49 years old)

‘… they [*nurses*] do not really take patients seriously … you have pain, you need medication, and no one is assisting … you go to the bathroom, you mess up yourself, you can remain for like that for four hours …’ (P5, SCI, male, 35 years old)

Family, friends, and community attitudes negatively affect patients’ care pathways:

‘I lost family in terms of now they don’t discriminate me but it’s kind of like they distance themselves …’ (P7, SCI, male, 32 years old)

#### Personal factors: Limitations in function

Patients described challenges related to limitations in their physical functioning required in activities of daily living, as well as restrictions on participation in various areas:

‘… I am struggling with balance … I cannot do the Coloplast. So, it is the Coloplast is something that helps with saving on nappies… I have pressure sores …’ (P2, SCI, male, 41 years old)

‘… the challenges I had here, is to take myself to a toilet … only physical challenges … Like bathing, changing clothes on your own.’ (P18, Stroke, male, 52 years old)

#### Personal factors: Personal beliefs

Personal beliefs and traditions can adversely affect patients’ health in the care pathway:

‘… after injury we tried this … west medication and African medication. So, people they say if you can take a sand and put on the fire and then you put your feet … I burnt my feet …’ (P21, SCI, male, 46 years old)

‘… they do not adhere to HIV to the ARV’s … when they come here, they do not disclose to their families they hide medication … relapse …’ (HOD 3, Ivy, female, 55 years old)

#### Personal factors: Self-motivation

HODs reported that a lack of patient self-motivation limited progress in the rehabilitation programme. Self-motivated patients were more compliant with treatment:

‘… you find that the person is not accepting their condition, so it’s a very big barrier … you can push how far if the patient has not accepted her condition, it’s very difficult to reach out to this person …’ (HOD 4, Ella, female, 62 years old)

**FIGURE 4:**
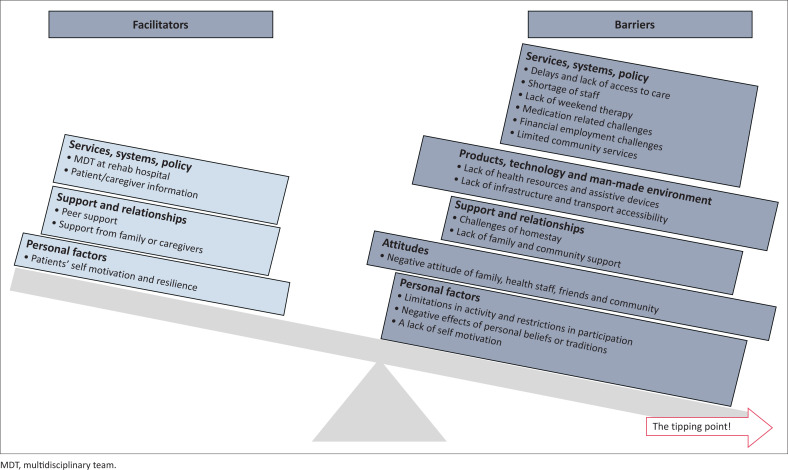
Barriers and facilitators.

Figure 4 is a summary of barriers and facilitators experienced in the care journey of PWND. This illustrates challenges in the health system where the barriers identified far outweigh the facilitators.

### Recommendations to improve health services and systems

**FIGURE 5:**
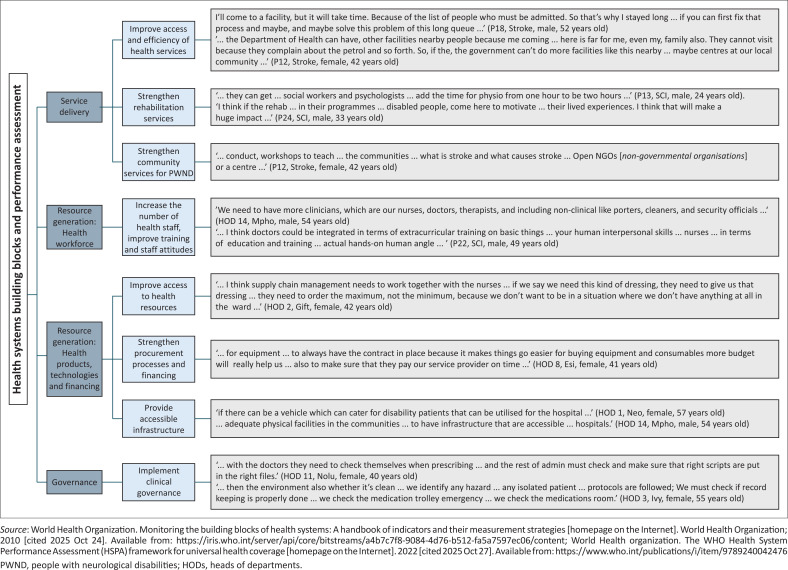
Participants’ recommendations categories and sub-categories.

The majority of participants recommended key improvements in the health system. These suggestions are categorised according to the WHO health systems building blocks and the health systems performance assessment for universal health coverage in Figure 5.^25,26^

## Discussion

The care pathway for PWND, such as stroke and SCI, was explored based on the lived experiences of patients and the work experience of various health professional HODs. As outlined in the study’s aims, several barriers and enablers were successfully identified throughout the care journey. Evidence suggests that there are health system challenges for PWND, as numerous environmental and personal-related barriers far outweigh the enablers reported. Participants also made suggestions on how to strengthen health services for PWND.

The study is unique in that it specifically targeted PWND at different stages over a care continuum to provide a more comprehensive exploration of the care journey. While the majority of previous studies were based at a single point in the care journey, they were not specific to PWND. The data suggest that while some patients were recently admitted to rehabilitation care, others were well into their rehabilitation programme or preparing for discharge, and various patients seen in the rehabilitation outpatient clinic had been attending for many years. Expert opinion was explored from every targeted health professional HOD in the hospital. These HODs had substantial experience in work and rehabilitation care, suggesting a deeper understanding. In summary, the diversity of demographic data, encompassing both patients and health professionals, enhances the comprehensiveness of the findings and supports broader applicability across PWND in rehabilitation settings.

### Environmental factors

The authors found that primary environmental barriers were reported in areas with poor health service delivery, health system inefficiencies, and limitations related to government directives. There was insufficient access to healthcare services, and in some instances, a complete lack of access, with rehabilitation care especially affected. Furthermore, participants reported various cases of inaccessible infrastructure and transport systems for PWND. Additional challenges identified included a shortage of health resources, significant barriers to securing employment following the onset of disability, and negative attitudes towards PWND exhibited by both family members or caregivers and healthcare professionals. These environmental factor barriers shared commonalities with previous South African studies.^33,34,35^ For example, Van Biljon et al.^35^ highlighted poor access to rehabilitation services for people with disabilities in public healthcare facilities, while Magaqa et al.^33^ identified a lack of rehabilitation services and resources at the primary health level. In this study, patients and HODS also reported delays in wheelchair access and repairs, a lack of medical supplies such as wound dressings and medicines, and a shortage of equipment. Heads of departments reported significant delays because of complex procurement processes and recommended procurement contracts that allow shorter turnaround times for the delivery of goods and services. Modisakeng et al.^34^ also identified some of these challenges in the South African public hospital procurement system.

Patients admitted to the rehabilitation hospital indicated that weekends were characterised by a lack of engagement in therapeutic interventions, resulting in periods of inactivity and associated feelings of boredom. The patients proposed that they could be more involved in physiotherapy or occupational therapy on weekends to facilitate a quicker recovery. This suggestion represents a novel perspective not documented in previous literature explored by the authors. The average length of stay of these patients at the rehabilitation hospital was 54 days; thus, implementing a patient therapy programme is recommended on weekends and public holidays with relevant in-hospital activities to optimise care and prevent risks in their physical and psychological health.^36,37^ Considering the reported limitations in the availability of healthcare resources and staff, the feasibility of the suggested weekend therapeutic rehabilitation may be limited.

Community care and integration for PWND were identified as severe environmental barriers because of the lack of specialised primary healthcare services, limited community awareness, inaccessibility to buildings or transport, a lack of care placement facilities, and a shortage of rehabilitation health facilities in the community. Although a few other South African studies support these findings,^17,33,38,39,40^ many were based on studies at one point in the care journey or included any patient requiring rehabilitation services. The evidence suggests that the South African Health Department needs to develop and implement a comprehensive community care package for PWND, in collaboration with relevant disability stakeholders and other government departments. Some components of this approach are also supported by the literature.^41,42^

Participants experienced various financial difficulties, and PWND were commonly dependent on social disability grants. The evidence suggests that the disability grant was not adequate to cover living expenses for PWND, and the approval of social grants was reported to be a drawn-out process.^10,43^ Difficulties in securing gainful employment post disability were yet another environmental barrier identified.^18^ In this study, wherein 73% of patients were unemployed, and many were unsure if they could return to work. It was found that the majority of these patients would also have to be reskilled for employment, as their jobs required manual labour, prior to their disability.^10,44^ Therefore, key participant recommendations to facilitate gainful employment for PWND are access to vocational rehabilitation services with emphasis on reskilling, accessibility to transport, adaptability at the workplace, and implementation of supporting government policies to accommodate PWND.^45,46^

Overall, within the health system, environmental barriers were also reported in the aspects of governance, care coordination, and health system responsiveness, where key health interventions can facilitate better outcomes.^47,48,49^ The evidence supports the participants’ suggestions for health system strengthening, which include improving clinical management, increasing budget allocations, improving the efficiency of procurement procedures, implementing skills training for health professionals, and implementing greater collaboration between governmental departments and disability stakeholders.

### Personal factors

The personal factors reported in this study highlighted the negative impact of limitations in a patient’s individual physical function that was dependent on the severity of their neurological-related disability. For example, patients with SCI experienced challenges in self-care and activities of daily living that are specific to their health impairment.^50^ Another personal factor was the impact of the patient’s own personal beliefs, where, for example, attempting traditional remedies or recreational activities resulted in burns to the lower limbs. Self-motivation has been described as a personal factor that reportedly influences how well a patient progresses through their rehabilitation programme. Heads of departments reported that patients who are not motivated take longer to reach the outcomes set in the rehabilitation programme. Participants recommended early and continuous patient and caregiver education and training related to personal factors, and the use of peer support.^51^

### Facilitators

The key enablers in the care pathway for PWND were rehabilitation care, an MDT approach, peer support programmes, and positive family or caregiver support.^12,52,53^ However, the evidence also suggests that these enablers were not consistent throughout the care pathway, and this influenced the quality of care. Therefore, the authors recommend, as also suggested by the participants, an MDT approach, education and training of patient, caregiver and health staff, the development of specialised services such as wound care, incontinence care, pain management, peer support, reintegration programmes, and vocational rehabilitation, with the provision of relevant health resources and budget support.^8,47,54^

## Conclusion

The care journey of PWND was explored to identify barriers, facilitators, and recommendations for strengthening the health system. The WHO, ICF, and Building Blocks of the Health System were used to categorise themes. The evidence shows that PWND experience numerous environmental and personal barriers, suggesting gaps within the health system. Key environmental barriers influencing the care pathway included limited or delayed access to care, inaccessible infrastructure and transport, a lack of health resources, financial and employment challenges, poor support, and attitudes. Personal factors were identified as the impact of limitations or restrictions on physical function, the patient’s own beliefs, and self-motivation. The key enablers identified in the care pathway for PWND were rehabilitation care, especially care at the rehabilitation hospital, an MDT approach, peer support programmes, and positive family or caregiver support. However, these enablers were not consistent throughout the different levels of care. Therefore, throughout the care journey, there is a need to improve accessibility, responsiveness, the quality of health treatment, and clinical governance. Community services and support for PWND are limited, and a more comprehensive care package is recommended. In addition, more intergovernmental collaboration and support from organisations supporting PWND are required to strengthen the care pathway, especially in rehabilitation care and community care.

### Limitations

The participants were recruited from individuals accessing services or working at the same Gauteng rehabilitation hospital, as part of a larger sequential multimethod PhD (Author Prisha Alakram-Khelawon). The rehabilitation hospital provides services for patients throughout the Gauteng province and across provinces, as there is a shortage of rehabilitation health facilities in the country (*South African National Health Act*, Categories of Hospitals). A more comprehensive study of PWND in community care is recommended, as is an exploration of the experiences of patients discharged from rehabilitation care who are living in other provinces.

### Implication for practice

Specialised health services for PWND are required for wound care, incontinence care, pain management, peer support, the implementation of MDTs, and rehabilitation care. There is a need to improve accessibility for PWND across infrastructure, transport, and community care. The role of clinical governance and the efficiency of hospital procurement procedures should be reviewed for PWND.
